# Effect of water on the glass transition of a potassium-magnesium carbonate melt

**DOI:** 10.1098/rsta.2022.0355

**Published:** 2023-10-16

**Authors:** Daniel Weidendorfer, Kai-Uwe Hess, Ruben M. Ruhekenya, Jürgen E. K. Schawe, Martin C. Wilding, Donald B. Dingwell

**Affiliations:** ^1^ Department of Earth and Environmental Sciences, LMU Munich, Theresienstrasse 41, 80333 Munich, Germany; ^2^ Analytical, Mettler-Toledo GmbH, Heuwinkelstrasse 3,8603 Nänikon, Switzerland; ^3^ Department of Materials, Laboratory of Metal Physics and Technology, ETH Zurich, 8093 Zurich, Switzerland; ^4^ UK Catalysis Hub, Research Complex at Harwell, Rutherford Appelton Laboratory, Harwell Campus, Harwell OX11 0DE, UK

**Keywords:** glass transition temperature, Gordon–Taylor analysis, potassium–magnesium carbonate, water, differential scanning calorimetry

## Abstract

Calorimetric measurements of the glass transition temperatures (*T*_g_) of hydrous carbonate melts are reported on a near-eutectic composition of 55 mol% K_2_CO_3_ – 45 mol% MgCO_3_ with up to 42 mol% bulk H_2_O dissolved in the carbonate melt. Hydrous melts were quenched from 750°C to transparent and crystal-free glasses and were subsequently analysed for water content before and after measuring *T*_g_ by high-sensitivity differential scanning calorimetry. The glass transition and limited fictive temperatures as a function of the water content were determined at 10 K/min cooling/heating rates resulting in *T*_g_ ranging from 245°C at nominally anhydrous conditions to 83°C in the presence of 42 mol% H_2_O in the glass. Through a generalized Gordon–Taylor analysis, the factors *k* (7.27), *k*_0_ (3.2) and the interaction parameter *A*_x_ (0.49) were derived. The limited fictive temperature of a hypothetically, zero water containing 55 mol% K_2_CO_3_ – 45 mol% MgCO_3_ glass is 232 ± 5°C (505 K). The high value of the interaction parameter *A* indicates strong specific molecular interactions between water and the carbonates in the glassy state and a large decrease in the excess enthalpy of mixing during the conversion of the glassy into the liquid state at the glass transition.

This article is part of the theme issue 'Exploring the length scales, timescales and chemistry of challenging materials (Part 1)'.

## 1. Introduction

Carbonate glass formation is restricted to chemical systems providing low-temperature eutectic melting regimes in respect to the quench rate-dependent temperatures crossing the glass transition [1]. Quantification of carbonate glass properties is of great importance in petrology to better constrain the ascend dynamics of mantle-derived carbonate melts [2,3] as well as the chemical interaction between low-viscous, highly mobile carbonate melts and the Earth lithosphere, which yield direct control on the globally operating carbon cycle between the various Earth reservoirs [4,5]. The extreme physical and chemical properties of carbonate melts (e.g. density, viscosity and conductivity) [6] are also underlining new development of more environmentally sustainable energy technologies such as molten carbonate fuel cells [7]. Amorphous carbonates are also important in catalysis, a synthetic form of the rare, amorphous copper hydroxycarbonate georgeite is used as a precursor to Zn-Cu catalysts used in methanol synthesis and in low-temperature water gas-shift reactions [8], and in this case, the activity of the catalysis is produced from (zincian) georgeite, which is superior to that produced from equivalent malachite. In the field of biomineralization, carbonate glasses play a significant role in respect to amorphous calcium carbonate as a precursor phase that enables organisms with control over the formation of vaterite, aragonite and calcite biominerals [9,10].

When compared to the list of well-studied multi-component silicate melt systems for their physico-chemical properties [11,–13], carbonate systems remain to date only poorly constrained, which ultimately relates to the poorly glass-forming nature of most carbonate melt systems. Glasses are formed when silicate, aluminate or borate melts are rapidly cooled through the quench rate-dependent calorimetric glass transition temperature. This transition temperature describes the quench rate-dependent transformation of undercooled melts to their glassy state due to an increase in viscosity that effectively suppresses nucleation of the mostly covalent network-forming melt components [14]. Glass formation is however not restricted to network-forming melts [15,–17], but also expands to organic, metallic or ionic salt systems including sulfates [18,19], nitrates [20] and carbonate melt systems [21]. From these glass-forming systems, carbonates are among the least studied chemistries due to their extremely high nucleation kinetics even at quench rates of 130–650°C/s in piston-cylinder or multi-anvil press static high-pressure experiments [22], which precludes the kinetic arrest of the melt within the supercooled glass transition regime. A vast majority of carbonate melt compositions therefore rarely form glasses upon quench from super-solidus conditions, but instead quench to compositionally heterogeneous mineral phases or mineral solid-solutions. The complexity of quench-induced nucleation [23] paired with rapid devitrification in poor glass-forming systems [24,–27] and the overall strongly hygroscopic nature of carbonates [23,28] on minute-to-hour-long time scales has challenged experimental investigation of the physico-chemical properties of carbonate melts at or above the glass transition temperatures.

There are three carbonate systems where glass formation has been reported since the early work of Eitel & Skaliks [29], who reported the first successful synthesis of carbonate glass by quenching a melt with approximately 50 : 50 mol% K_2_CO_3_–MgCO_3_ at 0.12 GPa. Carbonate glasses are currently limited to the systems of K_2_CO_3_–MgCO_3_ [21,23,29,31–], La(OH)_3_–Ca(OH)_2_–CaCO_3_–CaF_2_–BaSO_4_ [23,32] and to amorphous calcium–magnesium carbonate (see [33]). Dingwell *et al.* [21] provided the first calorimetric determination of the nominally anhydrous K_2_CO_3_–MgCO_3_ glass transition and proposed a highly non-Arrhenian viscosity–temperature dependence for fragile carbonate melts using the Vogel–Fulcher–Tammann fitting parameters. Performing high-pressure carbonate melting experiments bears the risk in contaminating the starting materials with water. The addition of water to the experimental charge may either take place before or after the synthesis due to the strong hygroscopic nature of Mg-bearing carbonates or hydration occurs during the experiment at high temperatures where hydrogen diffusion through the sample encapsulating noble metal partly reduces carbonate to graphite and H_2_O [28,34]. In order to determine the effect of water on the carbonate melt properties, we performed the first calorimetric observations of the glass transition temperatures of hydrous carbonate melts in the system K_2_CO_3_–MgCO_3_ with up to 42 mol% H_2_O (mole fraction expressed as 100 × *n*_H_2_O_/(*n*_K_2_CO_3__ + *n*_MgCO_3__ + *n*_H_2_O_)).

## 2. Materials and methods

### (a) Starting materials

All experiments were carried out on a near-eutectic composition consisting of 55 mol% K_2_CO_3_ and 45 mol% MgCO_3_ (table 1). Since reagent-grade magnesium carbonate contains significant traces of water, we used instead natural, high-purity magnesite as the source for MgCO_3_, which was pulverized and homogenized together with synthetic K_2_CO_3_ powder (Alfa Aesar Puratronic, 99.97%) in an agate mortar under ethanol to obtain a fine-powdered carbonate mixture. Energy-dispersive spectra of the natural magnesite confirmed chemical homogeneity with a maximum of 0.4 wt% FeO. Before homogenization, both carbonate powders were separately dried in ceramic crucibles at 250°C for 2 h before being permanently stored prior to capsule preparation in an oven at 105°C to avoid absorption of moisture from the atmosphere.

**Table 1.** Carbonate glass composition is expressed as wt% and mol% of K_2_CO_3_, MgCO_3_ and H_2_O.

	K_2_CO_3_	MgCO_3_	H_2_O	K_2_CO_3_	MgCO_3_	H_2_O
	wt%	wt%	wt%	mol%	mol%	mol%
KMG6-2	66.69	33.29	0.02	54.937	44.949	0.114
KMG5-2	66.59	33.24	0.17	54.414	44.520	1.066
KMG8-2	66.59	33.24	0.17	54.414	44.520	1.066
KMG9-2	66.59	33.24	0.17	54.414	44.520	1.066
KMG8	66.54	33.22	0.24	54.176	44.325	1.499
KMG4	66.17	33.03	0.80	52.331	42.816	4.854
KMG9	65.21	32.55	2.24	48.038	39.304	12.659
KMG5	63.54	31.72	4.74	41.833	34.227	23.940
KMG6	59.75	29.82	10.43	31.672	25.913	42.415

Mol% represent mole fractions of 100 × *n*_H_2_O, K_2_CO_3_, MgCO_3__/(*n*_K_2_CO_3__ + *n*_MgCO_3__ + *n*_H_2_O_).

### (b) Experimental methods

To load a capsule, a segment of annealed 3.0–4.0 mm OD Au tube was closed at one end with a triple-crimp and sealed by arc-welding. The crimped side of the Au capsule was then hammered flat before loading approximately 20–50 mg of dried K_2_CO_3_–MgCO_3_ powder into the noble metal container. Before closing the open end of the loaded Au capsule by arc-welding the triple-crimp at the top side of the capsule, the container was dried at 105°C to avoid atmospheric hydration. In experiments where deionized water was added to the powder-containing capsule, we added known quantities of H_2_O using a micro-syringe on a high-precision balance after drying out the powder-containing capsules again at 105°C for at least 30 min. After inserting H_2_O to the charges, the capsules were immediately welded shut using a PUK micro-welder.

Experiments were conducted at LMU Munich in a 12.7 mm diameter end-loaded piston cylinder using CaF_2_–graphite–MgO assemblies. For each experiment, one capsule was embedded in a crushable MgO sleeve and centred in the hotspot of the straight-walled graphite heater. The remaining space in the heater was filled with upper and lower crushable MgO cylinders including a graphite plug at the bottom. A two-hole mullite thermocouple protection tube was inserted in the drilled top MgO cylinder. Temperature was measured using a Pt_70_Rh_30_-Pt_94_Rh_6_ thermocouple (B-type), with no pressure correction on the emf. The welded thermocouple tip was separated by a thin MgO disc from the Au capsule. Pressure calibration of the piston-cylinder press revealed that no pressure correction is required for the CaF_2_–graphite–MgO assemblage at 1 GPa. Experiments were run at 1 GPa and 750°C. Each loaded assemblage was pressed cold to 1 GPa, isobarically heated to 750°C at a rate of 55°C/min and isothermally homogenized for 24 h. During temperature ramping, pressure was manually held constant by pumping hydraulic oil. Experiments were terminated after running 24 h at the run temperature by cutting the heating power, which allows for initial quench rates greater than 200°C s^-1^.

### (c) Analytical methods

Recovered Au capsules were cleaned of surrounding crushable MgO and subsequently opened on one side to extract the carbonate glass from the surrounding Au capsule material. In order to minimize post-experimental contamination of atmospheric moisture, the recovered glass was split into three parts using a stainless-steel razor blade, which then was immediately analysed (i) for textural homogeneity using a scanning electron microscope (SEM), (ii) for H_2_O using a combustion technique coupled to a thermal conductivity detector and to (iii) determine the glass transition temperature using differential scanning calorimetry (DSC). Some of the freshly recovered carbonate glass was analysed twice for its water content, before and immediately after the DSC measurement. Textural analyses of the run products followed immediately after sample recovery using a field-emission SEM (SU 5000 Schottky, Hitachi) operating in low-vacuum mode, which is equipped with an Oxford SDD energy-dispersive system.

DSC analyses were performed using a Netzsch DSC 404C. A single, transparent chip of the carbonate glass, of about 2–20 mg, was heated and cooled several times up to and from 280°C at a constant rate of 10 K min^−1^ in a Pt-crucible under a purging, high-purity Argon atmosphere (30 ml min^−1^). The temperature calibration was based on the melting points of Indium, Zinc, Ba-carbonate and Gold. By applying the temperature calibration, the absolute error of the measurement is ±1 K. The measurements were corrected for thermal drift.

Water contents of the carbonate glasses were quantitatively analysed by a ThermoScientific^TM^ FlashSmart^TM^ elemental analyser operating on a modified Dumas method where 2–5 mg of carbonate glass chips, enclosed in tin containers, are combusted in the presence of high-purity oxygen with helium acting as the carrier gas to an adjacent gas chromatograph. Hydrogen is detected by thermal conductivity and subsequently recalculated to weight percent H_2_O relative to the initial sample mass. The elemental analyser was calibrated on a BBOT standard and checked with secondary standards before and after measuring three replicates of each sample.

## 3. Results

### (a) High-pressure synthesis of carbonate glasses in the system K_2_CO_3_–MgCO_3_–H_2_O

**Figure 1.**
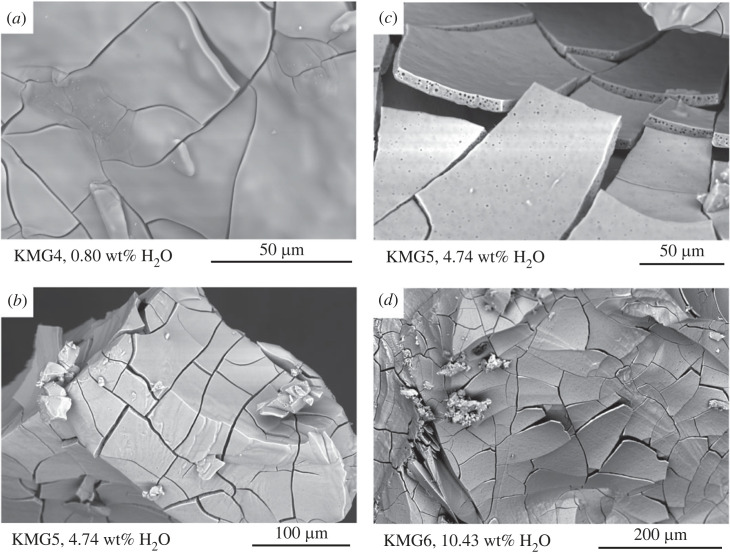
Back-scattered electron images of quenched carbonate glasses containing different bulk water contents. (*a*) Carbonate melt with 0.8 wt% H_2_O quenched to a vesicle-free transparent glass. (*b*) Increasing dissolved bulk water contents in the carbonate glass to more than 4 wt% results in a microlite-free glass that shows (sub)-micron-sized quench vesicles.(*c*,*d*) Mosaic texture is characteristic for post-experimental hydrous K-Mg-carbonate glasses.

After 24 h of thermal homogenization at 750°C, the nominally anhydrous K_2_CO_3_–MgCO_3_ melt (KMG 4, KMG8) was quenched to a clear and transparent carbonate glass when checked under an optical microscope. High-resolution SEM imaging confirmed formation of a crystal-free carbonate glass, without the presence of quench-induced vesiculation or microlite crystallization (figure 1).

In experiments where water was added to the K_2_CO_3_–MgCO_3_ starting composition, the obtained glasses were optically transparent and energy-dispersive analyses confirmed the glasses were compositionally homogeneous without the presence of microlites. Hydrous carbonate glasses are characterized by a split glass surface forming a mosaic texture with 5–30 µm large rectangular glass shards. Cross-sections of such hydrous glass shards display submicron-sized vesicles (figure 1). These small vesicles are homogeneously distributed in the outermost glass shards and are interpreted to result during experimental quench. When water containing charges were opened after the glass synthesis at 750°C, no excess fluid phase was, however, detected when checking for potential weight loss on a micro-balance.

### (b) Bulk water contents of carbonate glasses

Bulk water analyses of the synthesized carbonate glasses are particularly challenging when considering the hygroscopic nature of the K_2_CO_3_–MgCO_3_ starting material and the resultant carbonate glasses. A time series investigating for water absorption of K_2_CO_3_ powder exposed to air showed an uptake by the pre-dried carbonate powder of 2 wt% H_2_O after only 10 min. Similarly, the pre-dried 55K_2_CO_3_–45MgCO_3_ starting material mix used in our experiments absorbed up to 0.8 w% H_2_O in only 15 min at atmospheric conditions. Since the carbonate powder prior to loading into pre-experimental noble metal containers and the synthesized glasses are both sensitive to atmospheric moisture absorption, we have taken great care to minimize this risk by analysing the run charges directly after opening the sample containing capsules as well as immediately after the performed DSC analyses.

Nevertheless, the nominally anhydrous carbonate glasses of experiments KMG4 and KMG8 (no water initially added) yielded 0.8 and 0.24 wt% H_2_O, respectively (tables 1 and 2). The uptake of atmospheric moisture is hence unavoidable in such strongly hygroscopic chemical systems and even when keeping the time in between the individual pre- and post-experimental preparation steps to a minimum, sample hydration resulting in up to 0.8 wt% bulk H_2_O contents cannot entirely be circumvented. Hydrated carbonate glasses range in measured bulk H_2_O contents from 0.02 wt% (after DSC analysis) in sample KMG6-2 to water contents as high as 10.43 wt% (KMG6), or when recalculated on a molar basis the synthesized glasses yield between 0.11 and 42.42 mol% H_2_O (tables 1 and 2). Experiments KMG6 and KMG5 yielded 0.71 and 0.27 wt% less H_2_O than initially added prior to the high-pressure and high-temperature syntheses, which likely relates to subordinate fluid loss upon quenching the carbonate melts to temperatures below the glass transition as evidenced by the presence of submicron-sized quench vesicles (see §3a). Glasses of runs KMG5, KMG6, KMG8 and KMG9 were analysed before and after the performed DSC analyses and show a nearly complete dehydration after several heating and cooling intervals during thermal calorimetric measurements. KMG5, KMG8 and KMG9 glasses with initially 4.74, 0.24 and 2.24 wt% H_2_O, respectively, have dehydrated during DSC analyses to a residual water content in the carbonate glass at atmospheric conditions of 0.17 wt% H_2_O. Similarly, the initially 10.43 wt% H_2_O bearing KMG6 glass results in 0.02 wt% H_2_O (KMG6-2) after heating and cooling cycles through the supercooled liquid regime during DSC measurement (tables 1 and 2).

**Table 2.** Bulk water contents, peak and fictive glass transition temperatures (*T*_g_) of carbonate glasses.

	H_2_O	H_2_O^a^	carbonate	*T*_g,__peak_	*T*_g,__fictive_
units	wt%	mol%	wt fraction	K (corr.)	K
uncertainty (±)	0.05	0.005	0.005	1	3
KMG6-2	0.02	0.114	1.000	518	505
KMG5-2	0.17	1.066	0.998	516	502
KMG8-2	0.17	1.066	0.998	515	501
KMG9-2	0.17	1.066	0.998	515	501
KMG8	0.24	1.499	0.998	511	497
KMG4	0.80	4.854	0.992	497	484
KMG9	2.24	12.659	0.978	461	447
KMG5	4.74	23.940	0.953	417	403
KMG6	10.43	42.415	0.896	356	342
water^b^	100.00	100.000	0.000	—	136

^a^H_2_O mol% represents mole fraction of 100 × *n*_H_2_O_/(*n*_K_2_CO_3__ + *n*_MgCO_3__ + *n*_H_2_O_).

^b^[35,36].

### (c) Carbonate glass transition temperatures obtained from differential scanning calorimetry

**Figure 2.**
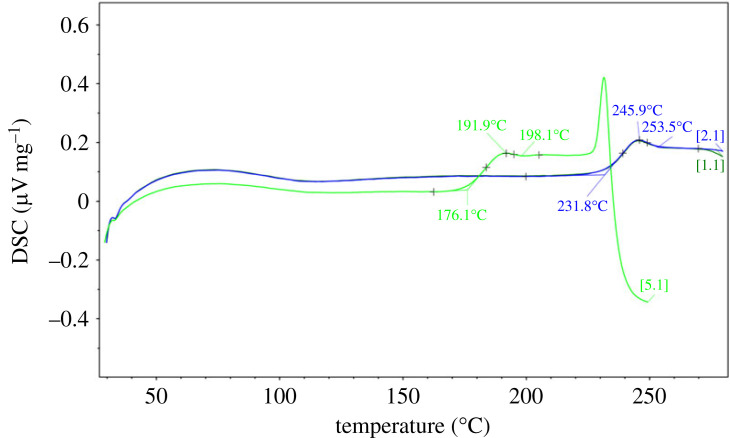
Raw signal DSC curves of carbonate glass sample KMG9 containing initially 2.24 wt% bulk H_2_O. The quenched glass was heated through the glass transition into the supercooled liquid regime where full relaxation occurs. A series of thermal cycles across the glass transition at a well-controlled heating rate (heating rate matches prior cooling rate of 10 K min^–1^) allows determination of the onset of the calorimetric glass transition, which is referred to as the limiting fictive temperature. (Online version in colour.)

**Figure 3.**
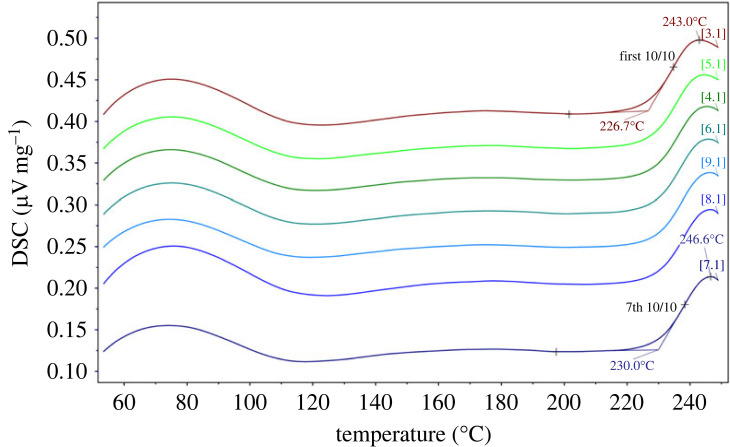
Raw signal DSC curves of carbonate glass sample KMG8 containing initially 0.24 wt% bulk H_2_O show below 120°C an artificial broad peak during specific heat recording (transition from isothermal conditions to a dynamic heating segment). The individual DSC curves show how successive reheating through the glass transition region shifts the glass transition temperature to higher values as a result of dehydration. (Online version in colour.)

The glass transition and limited fictive temperatures as a function of water content were determined at 10 K/min cooling/heating rates. Results of the DSC measurements are shown in figures 2 and 3 and are listed in table 2 for the K_2_CO_3_–MgCO_3_ glasses. The quenched glasses were heated through the glass transition into the supercooled liquid regime of up to 280°C (553 K), where the liquid is fully relaxed. The glass sample was then subjected to a series of thermal cycles comprising excursions across the glass transition at a well-controlled heating rate, with the heating rate matching to the prior cooling rate (10 K min^−1^; [37]). With this approach of matching cooling and heating rates, the so-called onset of the calorimetric glass transition (*T*_g,__onset_) corresponds to the limiting fictive temperature (*T*_g,__fictive_; [14,38,39]. Figures 2 and 3 illustrate that on successive reheating through the glass transition region, the glass transition temperature is shifted to higher values until a final value is reached. This effect can be triggered either by staying closely to the peak temperature for samples with initially low water contents (250°C for sample KMG8; figure 3) or by successively heating up to a temperature where degassing occurs in a single step (225°C for sample KMG9; figure 2). If the sample is completely degassed, the onset of crystallization occurs at about 270°C.

Raw signal DSC curves on a diagram plotting (µV mg^−1^) versus (uncorrected) temperature generally show at temperatures below 120°C an artificial broad peak during specific heat (*C*_p_) recording, which relates to a transition from isothermal conditions at room temperature to the dynamic heating segment of the DSC analysis (figure 3). In experiments with less than 4 wt% H_2_O, the low-temperature heating segment characterized by the beforementioned artefact is followed by a plateau representing the equilibrium state of the glass before the onset of the glass transition into the supercooled liquid state is calorimetrically detected. As the sample is heated at a rate of 10 K min^−1^, the temperature of the glass transition (taken as the peak position corresponding to the heat flow peak overshoot, hereafter referred to as *T*_g, peak_) decreases systematically with increasing dissolved bulk water content in the carbonate glass from 245°C (519 K) at 0.02 wt% H_2_O to 83°C (356 K) at 10.43 wt% H_2_O at a cooling rate of 10 K min^−1^. However, in hydrous glasses containing more than 4 wt% H_2_O only *T*_g,__peak_ could be identified and the glass transition on *T*_g_ onset was overlain by the broad artificial peak below 120°C. The difference between the *T*_g_ onset and *T*_g,__peak_ is on average 14 ± 2 K (independent of the initial amount of water). We could therefore estimate the extrapolated *T*_g_ onset (our proxy for *T*_g,__fictive_) for all samples, by applying the temperature correction (thermal lag) determined by the melting points of pure metals (subtracting of 4.5 K) and then further subtracting these 14 K. Temperature corrected data are listed in table 2.

## 4. Discussion

### (a) Carbonate glass transition temperature–bulk H_2_O relationship

**Figure 4.**
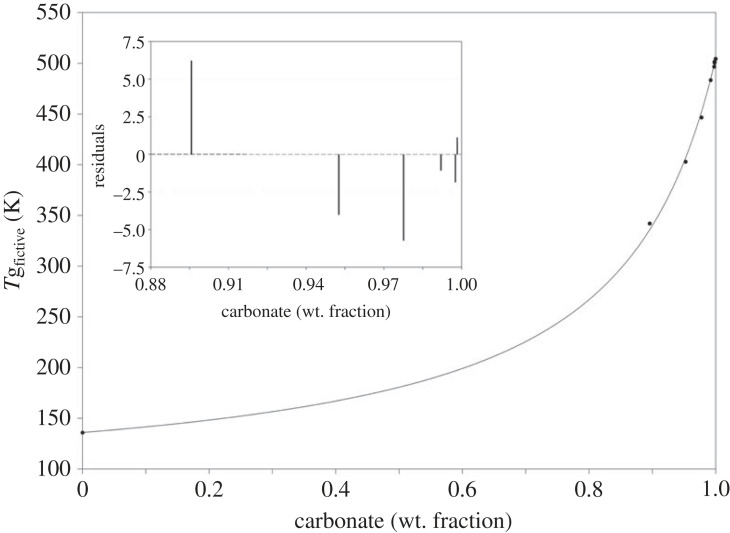
Plot shows relationship between limiting fictive temperature of the carbonate–water solution as a function of the carbonate weight fraction. Increasing water content in the carbonate glass results in decreasing glass transition temperatures. Data are fitted by Gordon–Taylor equation [40]. Glass transition temperature of pure water is taken from Hallbrucker *et al.* [35] and Capaccioli & Ngai [36]. Inlet displays residuals using the one-parametric fitting parameter.

Figure 4 displays the limiting fictive temperature of the carbonate-water solution as a function of the carbonate weight fraction content. The glass transition temperature of pure water is taken from literature [35,36]. The data show that increasing water content decreases the glass transition temperature of the amorphous mixture. Such a plasticizing effect can be described by the one-parametric Gordon–Taylor (GT) equation [40], which was derived for the case of athermal mixtures without specific molecular interaction between the different components. In some cases, this equation can be generalized and can be used also for strong molecular interactions [41]:

4.1 $$T_{\text{g}}=\frac{w_{\text{A}}\;T_{\text{g,}A}+\;kw_{\text{B}}\;T_{\text{g,}B}}{w_{\text{A}}+kw_{\text{B}}},$$

where *w*_A_ and *w*_B_ are the weight fraction of the components *A* and *B*, *T*_g,*A*_ and *T*_g,*B*_ are the glass transition temperatures of the pure components and *k* is the generalized GT parameter. We always define the components *A* and *B* in terms of the glass transition temperature with *T*_g,*A*_ > *T*_g,*B*_. According to [41], this parameter can be expressed using an interaction parameter *A* by

4.2 $$k=\frac{k_{\text{0}}+A}{1-A}.$$

The GT parameter for athermal mixtures, *k*_0_ = Δ*C*_p,*B*_/Δ*C*_p,*A*_, is the ratio of the intensities of the glass transitions of the pure components. This is the expected GT parameter if there are no specific interactions between the molecules of the two components. Deviations between the measured parameter *k* and the ideal parameter *k*_0_ are caused by molecular interactions characterized by the interaction parameter *A* with 0 ≤ *A* < 1. This concept was first developed to characterize the molecular clustering in systems forming hydrogen bonds [41]. In the case of carbohydrates containing systems, *A* depends on the most likely size of the metastable molecular clusters formed by hydrogen bonds. For the smallest cluster containing two molecules, the interaction parameter is *A* ≈ 0.2. Larger cluster sizes lead to higher values of *A* in the trehalose-water system, of *A* ≈ 0.51. This system forms large metastable clusters. Therefore, trehalose is often used to prevent freezing of water at low-temperature applications, e.g. cell preservation or freeze-drying. Since hydrogen bonds are relatively strong molecular interactions, we classify *A* as following in a first attempt:

— *A* = 0: no specific molecular interaction between the different kinds of molecules.
— 0 < *A* < 0.2: weak specific molecular interactions.
— 0.2 ≤ *A* ≤ 0.6: strong specific molecular interactions.
— 0.6 < *A*: very strong specific molecular interactions.

This classification can be improved by further investigations of amorphous mixtures with various molecular interactions. The model developed in [41] can be generalized in order to describe the conditions in different systems without cluster formation (see electronic supplementary material, Appendix). The result shows that the interaction parameter *A* is linked to the difference of the excess enthalpy of mixing between the liquid and the glassy state, Δ*h_e_*.

In our nomenclature, the carbonate mixture is component *A* and water component *B*. The parameter *k* = 7.27 is determined by curve fitting (figure 4). For the fit, we used the value of 505 K (sample KMG6-2) as the value for *T*_g,*A*_. Figure 4 shows the residual plot of the fit. All glass transition temperatures (*T*_g,__fictive_) can be fitted within ±  6 K; the *r*^2^ is 0.999.

It is Δ*C*_p,B _= 1.94 J/(gK) for amorphous water [42]. For the intensity of the glass transition of the carbonate mixture, we determined Δ*C*_p,*A* _= (0.60 ± 0.06) J/(gK). This leads to *k*_0_ = 3.2 and *A* = 0.49. The difference between *k* and *k*_0_ is quite large. The interaction parameter *A* is close to the system water-trehalose indicating strong specific molecular interactions between water and the carbonates in the glassy state. The relatively high value of *A* could also indicate a relatively large decrease in the excess enthalpy of mixing during the conversion of the glassy state into the liquid state at the glass transition. This may explain the water loss after the glass transition.

### (b) Effective water contents in high-pressure carbonate glass synthesis

The formation of glasses in carbonate systems is problematic, since carbonates are ionic liquids and seem to not undergo polymerization as seen in glass-forming silicate systems. Spontaneous crystallization of carbonate melts may relate to the overall low activation energies of carbonate nucleation and crystal growth rates [23]. An exception is the K_2_CO_3_–MgCO_3_ system where carbonate glasses can be formed at quench rates achievable with static high-pressure equipment [21,29,31,32,43–46]. The first potassium-magnesium carbonate glass was reported by Eitel & Skaliks [29] at 0.12 GPa near the eutectic at 57 mol% K_2_CO_3_ – 43 mol% MgCO_3_. In the same chemical system, Arefiev *et al*. [47] and Shatskiy *et al*. [48] performed phase equilibria experiments at 3 GPa and 6 GPa, respectively, however, no carbonate glass was quenched at these high pressures. Our water-bearing K_2_CO_3_–MgCO_3_ glasses synthesized at 1 GPa demonstrate that carbonate glass formation is not restricted to a low-pressure regime (0.1–0.12 GPa; e.g. [21,29], even though at pressures greater than 3 GPa the eutectic is shifted to the magnesium-rich side of the binary phase diagram and even splits at 6 GPa into two eutectics that are separated from each other by a thermal divide at 50 mol% MgCO_3_ [48], which altogether may yield control of the compositional space where carbonate glass formation occurs.

Determination of the truly anhydrous carbonate glass transition temperature is experimentally nearly impossible to achieve due to the ubiquitous hygroscopic nature of the K_2_CO_3_–MgCO_3_ near-eutectic composition. Therefore, our experimental and analytical approach strenuously aimed at minimizing atmospheric moisture absorption during pre- and post-experimental sample preparation, which ultimately allowed determining the closest possible experimental approximation of the ‘dry’ glass transition temperature (245°C at 0.02 wt% H_2_O, sample KMG6-2). Furthermore, presumably undetected water contents of previous reported nominally anhydrous carbonate glasses [21,29,31,32,43–46] can now be reconstructed through equation (4.1) using the one-parametric GT fit to our obtained *T*_g_–H_2_O relationship (figure 4). According to the established *T*_g_–H_2_O relationship, the recently nominally anhydrous *T*_g,__peak_ of 230.5°C (*T*_g,__fictive _= 216.5°C; 490 K) reported by Dingwell *et al*. [21] for carbonate glasses quenched from melts of near-eutectic composition (55 mol% K_2_CO_3_ – 45 mol% MgCO_3_) suggest the presence of approximately 0.5 wt% H_2_O during their glass synthesis at 0.1 GPa.

### (c) The water content of amorphous carbonates

The water content of amorphous carbonates strongly influences their crystallization. The distinction between glass and amorphous materials is becoming increasingly blurred (see [33]) when thermal analysis using rapid heating rates is used, and there is a complex relationship between dehydration reactions, glass transition onset and crystallization. As noted above, the rare amorphous, copper hydroxycarbonate georgeite is an important catalyst precursor [49]. This material can be synthesized [50] readily, calcined and reduced to form Cu- and, in the case of Zn-bearing samples ZnO-Cu-catalysts. The activity of the catalysts depends on the georgeite precursor and the formation of malachite, the latter is supressed when a super-critical CO_2_ route is used [8,50] rather than co-precipitation routes, where georgeite occurs transiently. The rare occurrence of georgeite in nature with malachite formation is also likely to reflect the water content of the amorphous form and, if indeed glass transitions can be identified in these rare amorphous minerals, the dependence of the glass transition on water content will strongly influence the dehydration steps prior to catalyst formation. Furthermore, the formation of hydroxycarbonates via geological processes (hydrothermal processes) when compared with the chemical synthesis routes such as super-critical CO_2_ and formation and stability of intermediate amorphous forms is also likely to reflect the influence of water content on the glass transition.

## 5. Conclusion

Similar to hydrous silicate and borate glasses [11–13], the glass transition temperature of the studied hydrous carbonate glasses progressively shifts to lower temperatures with increasing water weight fraction. In direct comparison to hydrous silicate glasses, the synthesized carbonate glasses from this study yield significantly lower *T*_g_ values of 245°C at near anhydrous conditions to values as low as 83°C in the presence of 42 mol% H_2_O. Such extremely low K_2_CO_3_–MgCO_3_–H_2_O glass transition temperatures represent to the best of our knowledge the lowest values for any petrologically relevant melt composition and may yield important further implications for hydrothermally active subsurface crustal environments including carbonate-bearing materials derived through sedimentary, magmatic or biomineralization processes.

## Data Availability

All data is listed in tables 1 and 2 and visualized in figures 1–4. Additional information is provided in the electronic supplementary material [51].
